# Increasing Support for Contraception as HIV Prevention: Stakeholder Mapping to Identify Influential Individuals and Their Perceptions

**DOI:** 10.1371/journal.pone.0010781

**Published:** 2010-05-24

**Authors:** Tricia Petruney, Sarah V. Harlan, Michele Lanham, Elizabeth T. Robinson

**Affiliations:** 1 Research Utilization, Family Health International, Research Triangle Park, North Carolina, United States of America; 2 Information Programs, Family Health International, Research Triangle Park, North Carolina, United States of America; 3 Behavioral and Biomedical Research, Family Health International, Research Triangle Park, North Carolina, United States of America; CIET, Canada

## Abstract

**Background:**

Voluntary contraceptive use by HIV-positive women currently prevents more HIV-positive births, at a lower cost, than anti-retroviral drug (ARV) regimens. Despite this evidence, most prevention of mother-to-child transmission (PMTCT) programs focus solely on providing ARV prophylaxis to pregnant women and rarely include the prevention of unintended pregnancies among HIV-positive women.

**Methodology/Principal Findings:**

To strengthen support for family planning as HIV prevention, we systematically identified key individuals in the field of international HIV/AIDS—those who could potentially influence the issue—and sought to determine their perceptions of barriers to and facilitators for implementing this PMTCT strategy. We used a criteria-based approach to determine which HIV/AIDS stakeholders have the most significant impact on HIV/AIDS research, programs, funding and policy and stratified purposive sampling to conduct interviews with a subset of these individuals. The interview findings pointed to obstacles to strengthening linkages between family planning and HIV/AIDS, including the need for: resources to integrate family planning and HIV services, infrastructure or capacity to provide integrated services at the facility level, national leadership and coordination, and targeted advocacy to key decision-makers.

**Conclusions/Significance:**

The individuals we identified as having regional or international influence in the field of HIV/AIDS have the ability to leverage an increasingly conducive funding environment and a growing evidence base to address the policy, programmatic and operational challenges to integrating family planning with HIV/AIDS. Fostering greater support for implementing contraception for HIV prevention will require the dedication, collaboration and coordination of many such actors. Our findings can inform a targeted advocacy campaign.

## Introduction

**Figure 1:**
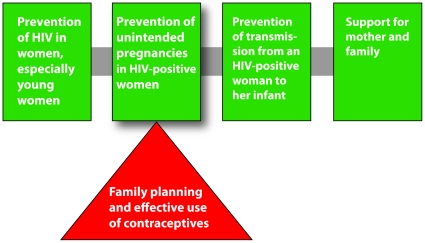
WHO/UNAIDS strategy for the prevention of mother-to-child transmission of HIV (see [1]). Triangle added for emphasis by Family Health International.

Ninety percent of new HIV infections among children under age 15 are a result of mother-to-child transmission [1]. To address this problem, the World Health Organization (WHO) and the United Nations Joint Programme on HIV/AIDS (UNAIDS) outlined a four-element strategy for the prevention of mother-to-child transmission (PMTCT) of HIV (see Fig. 1).

Although linking family planning and HIV services is generally considered feasible and effective [2], [3], [4] and despite the fact that the contributions of contraception to reducing MTCT have been well documented [5], [6], [7], the vast majority of PMTCT programs continue to focus almost exclusively on providing antiretroviral (ARV) prophylaxis to pregnant mothers and infants [8]. The percentage of HIV-positive pregnant women who received ARV treatment to prevent vertical transmission reached an average of 45% in 2008, up from 35% in 2007 [9]. Indeed, nearly 240,000 infant infections have been averted through ARV regimens cumulatively over the past five years [10]. The steady progress made in reaching pregnant women with ARV interventions is a significant public health achievement. In comparison, however, models project that *current* contraceptive use may already be preventing more than 220,000 HIV-positive births *per year* in countries hardest hit by the HIV epidemic [11], [12]. By these estimates, voluntary contraceptive use prevents more infants from becoming infected with HIV than the more common intervention of treating the mother and infant with ARVs. As these estimates are based on current (low) contraceptive use in developing countries, and as several studies have shown that women who know they have HIV are less likely than other women to report wanting additional children [13], [14], [15], efforts to alleviate current unmet need for family planning (often high among HIV-infected women [16], [17], [18]) could substantially increase the number of HIV-positive births averted.

We implemented a project based on the theory that support from key influential individuals is critical to widespread adoption of a new or underused idea or practice [19], [20]. Such individuals act as opinion leaders, and since they play key roles in influencing HIV/AIDS decision-making and strategic planning, may speed the diffusion of new ideas or practices [21], [22], [23]. Based on these theories our project goals were: 1) to identify the most influential individuals in the international HIV/AIDS field–i.e., those who currently inform HIV/AIDS funding, policy, programming and research; 2) to determine their perceptions of the barriers to supporting or implementing the strategy and factors that might facilitate its adoption; and 3) to reach the influential individuals identified in the first step of the process with a communications intervention that addressed identified facilitators and barriers.

This paper focuses on the first two goals. We used criteria-based searches to identify key opinion leaders in HIV/AIDS, and through key informant interviews we explored their knowledge of contraception as an effective PMCT strategy, and their perceived facilitators and barriers to implementing element 2 of a comprehensive PMTCT program.

## Methods

### Stakeholder Identification

To identify opinion leaders in HIV/AIDS we adapted a stakeholder analysis methodology that focused on identifying influential individuals [24]. We defined stakeholders as “all parties who will be affected by or will affect” the issue [25]. We postulated that different types of HIV stakeholders would be necessary to “tip” [20] the uptake of contraception as an HIV prevention strategy. We focused on identifying stakeholders among groups that have the *most direct impact* on HIV/AIDS research, programs, funding and policy: researchers, program managers, donors, policy makers, advocates, and other public figures.

We searched online information products–published research, conference presentations, grants, media digests, and lists of key staff/directors of major HIV and AIDS organizations–to identify and create a list of key HIV/AIDS stakeholders. To ensure that developing country stakeholders were included, we supplemented our global searches with additional searches in six countries: Kenya, Nigeria, Uganda, South Africa, Tanzania, and India.

To identify key researchers, we searched online journals (via PubMed and Popline databases), conference presentations, and NIH grants. Due to the high volume of HIV researchers listed in PubMed, we chose to add individuals to the list only if they had published research on PMTCT.

To find key HIV program planners and managers, we searched for organizations implementing work funded by the Bill & Melinda Gates Foundation, the President's Emergency Plan for AIDS Relief (PEPFAR), the World Bank, and the Global Fund for AIDS, Tuberculosis and Malaria (GFATM). We added principal grant recipients, team leaders, and others in leadership positions to the list.

To identify public figures in HIV/AIDS that may not have been found in our other searches, we reviewed two years of *Kaiser Daily HIV/AIDS Reports* (an email newsletter and online digest). To narrow down the list of individuals included in these news reports, we only added individuals to the list if they were mentioned or quoted in a news summary about PMTCT, and we excluded current heads of state.

Finally, we conducted internet searches to find lists of managers, directors, and other high-level staff at major global HIV/AIDS donor organizations and normative bodies (GFATM; World Bank; WHO; UNAIDS; PEPFAR; Elizabeth Glaser Pediatric AIDS Foundation; and the Bill & Melinda Gates Foundation), and the leaders and members of major international HIV and AIDS task forces, working groups, research organizations, and advocacy organizations (The Global HIV Prevention Working Group; International AIDS Alliance; the African Medical and Research Foundation [AMREF]; The American Foundation for AIDS Research [amfAR]; the Center for HIV-AIDS Vaccine Immunology [CHAVI]; and the National Institute of Allergy and Infectious Diseases [NIAID]). We included all of these global leaders in our list.

### Key informant interviews

#### Selection of interviewees

For the process of both identifying interviewees *and* deciding whom to target with individualized messages about contraception for HIV prevention (i.e., the communications intervention), we sought to narrow down the larger list to identify the *most* influential leaders. To select these individuals, we used stratified purposive sampling [26].

To ensure that key informants represented the views of the larger group that we identified, we based our selections on the overall geographic distribution of individuals, choosing from both the global and country-level lists. Furthermore, we wanted the interview responses to reflect the range of individual characteristics and societal roles of these opinion leaders, so we selected individuals who represented a variety of organizations and institutions (maximum variation sampling). Finally, individuals who appeared in more than one data search were included in our list of interviewees (intensity sampling). We hypothesized that these individuals would be “information-rich,” and would have detailed knowledge of the topic [26], [27]. They appeared in multiple data searches which suggests that they have multiple spheres of influence, and thus could provide valuable insights into what types of messaging would be effective for a communications and advocacy campaign.

We selected forty-eight individuals for interviews based on these maximum variation and intensity sampling techniques. However, due to the high-profile–and thus hard-to-reach–status of some of the individuals on our list, we narrowed this list down further to 24 individuals on the list who were known by colleagues, were easily reachable, or otherwise the most likely to respond (opportunistic sampling) [28]. We conducted in-depth interviews with 22 out of 24 individuals who responded.

#### Interview format and analysis

We conducted semi-structured qualitative interviews in English, either in person or by phone. Interviews ranged from 10 minutes to 30 minutes in length. All interviews were tape recorded and then transcribed verbatim. Participants were not reimbursed or given any incentives for participating in interviews.

Each interview explored the following topics:

- The participant's general opinion about contraception for PMTCT
- Any professional work he/she was currently doing that supported contraception for PMTCT
- What resources his/her organization might need to more actively support implementation of such activities
- What factors he/she believed could facilitate or provide a more prominent place for contraception among PMTCT efforts
- What objections he/she has perceived among those who do *not* support integrating contraceptive services into HIV prevention and PMTCT efforts
- Who he/she thought could have a strong influence over others if they were to promote contraception for the prevention of HIV
- Where he/she accesses information about HIV and/or contraception, and which communication channels most influence his/her decisions

All parts of the interviews were reviewed, but we focused our coding and analysis on: opinions about contraception for PMTCT; opinions about barriers/facilitators of implementing integrated family planning/HIV services; and communication channels.

Two analysts read through several transcripts and independently identified a list of emerging ideas, which they grouped into 12 broad themes. The two individuals worked together to develop a codebook. Each theme was given a code. They independently coded each transcript. Due to the small data set, the analysts coded the data by hand and no software was used. Using an iterative process, they altered the codebook as new themes emerged.

The two individuals met to compare how they coded each transcript. Any discrepancies between the two coders were resolved through discussion. Since all discrepancies were resolved, no inter-reliability metrics were needed. The analysts calculated the total number of interviews in which each theme was mentioned (for the entire sample), and then compiled separate totals for country-level and global-level interviews. They also performed a thematic analysis of the interviews, examining the major themes and ideas that arose in each interview [28], including an analysis of the differences between the answers of interviewees working at the country level and those working at the global level. Finally, they ran frequencies for the question about information and communication channels.

## Results

### Stakeholder Identification

Through our searches we generated a list of 467 HIV stakeholders. All of our target groups for the future communications intervention were represented in this list: researchers, program managers, policymakers, donors, advocates, and other public figures. Forty-eight percent were based in Africa, 28% in North America, 13% in Europe, 10% in Asia, 0.6% in Latin America and the Caribbean, and 0.4% in Australia.

The number of stakeholders identified by each of our data search methods is shown in Table 1.

**Table 1:** Number of stakeholders found by data search.

Data Search	# of stakeholders found
Published research, conference presentations, NIH PMTCT grants	117
HIV program planners and managers (Global Fund, PEPFAR, World Bank, Gates Foundation)	131
*Kaiser Daily HIV/AIDS Reports*	31
Leadership of major HIV/AIDS organizations, normative bodies, international task forces, working groups, research organizations and advocacy groups	218

(Note: The sum equals a higher number than the final stakeholder total due to several individuals who appeared in more than one of the four searches. The total number of stakeholders was reached after removing duplicate individuals.)

### Key informant interviews

After applying stratified purposive sampling, forty-eight individuals were eligible to be interviewed. The 22 interviewees chosen were the most accessible and available. The geographic distribution of the 22 key informants (12 from Africa; 9 from the US; 1 from Europe) and the male to female ratio (15 male; 7 female) were similar to the broader group of stakeholders. The group of interviewees included 11 researchers and scientists, 5 national-level health officials, 3 international donors, 2 advocates, and 1 program manager. Five of the key informants sit on the high-level *Global HIV Prevention Working Group*.

Although we asked about participants' perceptions of contraception as a PMTCT strategy, respondents often talked about family planning and HIV/AIDS integration more broadly since strengthening linkages between the two is how contraception for HIV prevention is typically operationalized (in addition to generally strengthening family planning programs for all women in high HIV prevalence settings) [29]. Although we asked about both barriers to and facilitators for implementing family planning as HIV prevention, the responses we received focused on the former.

Table 2 summarizes the themes related to barriers to implementing family planning/HIV integration that emerged from the interviews and the number of stakeholders who mentioned each theme. This table presents the results for all respondents together, and also offers a comparison of the themes emerging from country-level and global-level stakeholders' interviews; we classified 10 interviewees as working primarily at the global level, and 12 as working primarily at the country level.

**Table 2:** Key interview findings by theme.

Theme	# of overall informants mentioning theme (out of 22)	# of global-level informants mentioning theme (out of 10)	# of country-level informants mentioning theme (out of 12)
Lack of resources to link FP and HIV/AIDS services	20	9	11
Lack of infrastructure or capacity to provide integrated services at the facility level	18	7	11
Lack of national-level leadership or coordination	16	4	12
Lack of targeted advocacy to decision-makers	16	7	9
Lack of knowledge and/or understanding of FP's contributions to HIV prevention	15	7	8
Separation of or competing resources for FP and HIV/AIDS	15	8	7
Cultural or religious resistance to family planning	12	5	7
Family planning or HIV/AIDS program managers and service providers seeing the responsibility as lying elsewhere	12	7	5
Separation of family planning and HIV/AIDS programs	12	8	4
Political resistance from decision-makers to family planning	10	4	6
Lack of global-level leadership or coordination	8	5	3
Lack of influential individuals as ‘champions’	7	4	3
Separation of family planning and HIV/AIDS policies	7	3	4
Lack of monitoring and evaluation systems for integrated services	5	3	2
Indifference at policy or donor levels toward strengthening linkages between FP and HIV/AIDS	3	1	2
Lack of visibility in published literature or technical conferences on the benefits of integrating FP and HIV/AIDS services	3	3	0

Overall, the most common theme in the interviews was a ‘lack of resources to link family planning and HIV/AIDS services’, which was mentioned as a barrier to implementing integrated family planning and HIV/AIDS services by 20 participants. Interviewees usually used the term “resources” to refer to funding for services generally (either family planning or HIV/AIDS); however, human resources, contraceptive commodities, and clinic supplies were also discussed.

Many participants mentioned that while HIV programs may benefit from integrating family planning with other clinical services, the overall lack of funding for existing services makes implementation difficult. As the director of one infectious disease institute in Africa explained, “*[A]lthough we want to incorporate it* [family planning] *into a* [HIV/AIDS] *clinic we would need…additional staff to help in the clinics where they don't have enough resources for anything.”* One HIV researcher said, “*Organizations follow the money,”* so the most effective way to strengthen linkages between family planning and HIV is to *“encourage funding organizations to make this a priority.*”

The second most common theme, discussed by 18 participants, was the ‘lack of infrastructure or capacity to provide integrated services at the facility level’. Each of these interviewees appreciated the need for the provision of both HIV and family planning services within the same physical structure. Most participants listed specific constraints that prevent this from being a norm. One senior AIDS official from a ministry of health in East Africa said, *“We need support in training health workers to give them skills, but also for supervision to make sure that integration is properly done, especially maintaining the quality of care.”* This lack of training and support for health workers was mentioned often as an obstacle to implementing integrated family planning/HIV programming. An interviewee from Uganda said, *“Capacity building and training the service providers to be able to handle these services* [is needed]*…if it is the same health worker who is providing counseling and services for HIV prevention, and he or she is trained in family planning services, then integration becomes easy.”*

Sixteen participants discussed the ‘lack of national-level leadership or coordination’ as a barrier. A participant from Uganda said, *“There is need for leadership to be able to guide the integration process so that if you mobilize resources for–say–HIV/AIDS, then the leadership should be on board in terms of guiding this money… to advance the cause of family planning.”* Some informants proposed that more national coordination or “ownership” of the issue from ministries of health and other national officials could improve implementation by providing a platform on which to build bridges between family planning and HIV programs and carry out existing supportive policies. Another interviewee from Uganda noted, *“Advocacy* [is needed] *within the Ministry of Health itself ultimately. It is the Ministry of Health who are responsible for health care.”*

A ‘lack of targeted advocacy to key decision-makers’ was also mentioned by sixteen participants. They noted the need for advocacy aimed at stakeholders across the spectrum, including global policymakers, national program managers, and individual service providers. Many participants recommended that advocacy be targeted at international donors, including a senior official from an international foundation who said, *“What it is going to take is some advocacy to make it happen. I'm always optimistic that funding organizations can be encouraged to do quite a bit. They are quite open to it, so I think it is probably a matter of advocates getting them interested in it.”*

Fifteen participants mentioned ‘lack of knowledge and/or understanding’ of contraception's contributions to HIV prevention and the benefits of family planning/HIV integration among all levels of stakeholders as a barrier. Most interviewees who mentioned this linked it with the need for advocacy to remedy the lack of understanding.

The separate funding streams for family planning and HIV/AIDS programming and services, from donors and within national governments, were also mentioned by fifteen participants as a barrier to integrated service provision. *“Often what happens, because the funding streams are somewhat artificially divided, is health care personnel are trained in HIV/AIDS prevention care or treatment and then they are trained, perhaps separately, around family planning. … The training cycles are not integrated,”* said the top official at an international HIV/AIDS foundation. This challenge was often described as a reason why program planners are not able to design comprehensive services, and also as a contributor to overlapping efforts and inefficient use of resources. With regard to strict HIV programming, an HIV researcher explained that the prevailing thinking is “*This is an HIV initiative, not a family planning initiative, and we are not going to use HIV money for these other things. This does not make any sense because if* [family planning] *has an HIV impact then it should be required of an HIV program.”*

Further, some participants suggested that this separation of resources may be the cause of competition or tension between the reproductive health and HIV/AIDS fields over which programs are responsible for what services, especially when resources are not available to create bi-directional integrated programs. This was illustrated when a staffer at WHO stated, *“There is a tendency*… [in] *some parts of the HIV community to see their primary area of work as being rather more narrowly defined HIV services, and saying, ‘Family planning is important, sure, but that's not our responsibility.’ …you'll find that reproductive health people are struggling with family planning service generally and they kind of feel like they don't have the means* [to offer HIV services] *…if a woman has HIV, programs are available and they are so well resourced so surely they* [HIV providers] *should be doing it.”*

Overall, similar themes were mentioned by both country-level and global stakeholders (see Table 2). However, we found some differences in what the two groups identified as important.

Those working at the global level focused more on macro-level funding, global policy and overall trends than did interviewees working at the country level. The theme ‘separation of family planning and HIV/AIDS programming’ was more common in these interviews than among the country-level participants. Global stakeholders also pointed out the tendency of both family planning *and* HIV/AIDS professionals to avoid responsibility for implementing integrated programs (the theme ‘family planning or HIV/AIDS program managers and service providers see the responsibility as lying elsewhere’).

Compared to global level participants, country-level interviewees focused more on the specifics of implementing family planning and HIV integration. The most common theme among this group was the ‘lack of national leadership or coordination’, which was mentioned by all country-level informants. Additionally, more participants at the country-level than the global-level mentioned the ‘lack of infrastructure or capacity to provide integrated services at the facility level’ as a barrier to integrating family planning and HIV programs.

Regarding which communications channels the participants use to make decisions about HIV/AIDS research, funding, policy, or programming, all 22 key informants said that they take into account information received at meetings and conferences; 18 of the 22 responded that they use information from print publications, online publications, and other colleagues; 17 informants said that they use information found in emails; and 10 said the same for listservs (though listservs and email were both more important to those working at the global level than at the country level). Television and radio were not seen as important sources of information for the majority of the participants. In response to a question about which information source *most* influences their decisions, 15 of the 22 interviewees said either print or online publications; 7 individuals stated that conferences/meetings were the most important source of information for them.

## Discussion

Funding for HIV/AIDS continues to increase [30] as donor assistance for family planning essentially stagnates and continues to fall short of meeting needs [31]. At the same time, despite continued growth in international policy support for linking family planning and HIV/AIDS, parallel funding streams and programming structures persist at the global and national levels [32]. This has largely remained the case despite clear evidence of the effectiveness and cost-efficiency of contraception's contributions to PMTCT. Indeed, the current narrow focus of PMTCT programs is only one example of how disease-specific global health funding, particularly for HIV/AIDS, can exacerbate the verticalization of recipient countries' health systems [33]. With limited human and financial resources to establish or strengthen a primary care model and improve the capacity of health care workers to provide comprehensive care packages (such as integrated family planning and HIV/AIDS), and without strong national or donor mandates to do so, programs continue struggling to deliver integrated services.

The results of our key informant interviews with opinion leaders in HIV/AIDS substantiated these barriers and constraints. Regardless of their professional affiliation, most of the stakeholder informants told us the biggest challenges to implementing element 2 of WHO's PMTCT strategy were restricted or siloed funding and the need to improve health infrastructure and human capacity to provide better services. It is worth noting that our interviews took place in 2008, before the U.S. presidential election and subsequent administration change. Since that time, a growing number of USAID bilateral agreements are encouraging integration by combining funding for HIV/AIDS and family planning into single health programs, and global HIV/AIDS funding mechanisms like PEPFAR and the Global Fund are becoming increasingly supportive of family planning services as essential components of HIV/AIDS programs.

During our interviews, participants recommended directing more advocacy at international donors to counter the lack of and parallel nature of family planning and HIV/AIDS funding, As the funding environment continues to improve, however, advocacy and communication campaigns will eventually need to shift toward national decision-makers and program planners to improve their ability to leverage these new and less restrictive funding opportunities. To strengthen national-level coordination, participants suggested that countries take more ownership over the issue of harmonizing planning for funding, programming and training structures between the two fields. The importance of establishing close partnerships between the family planning and HIV/AIDS sectors was mentioned by several interviewees as a way to overcome the challenges of parallel programming.

Few participants mentioned the ‘lack of visibility in published literature or technical conferences’ as a main reason why this HIV prevention strategy has not been more prominent. At the same time, the theme of ‘lack of knowledge and understanding’ came up frequently, suggesting that the evidence regarding family planning's contribution to HIV prevention does in fact need to be disseminated more broadly. Furthermore, as the evidence base for effective models of family planning and HIV/AIDS integration continues to grow, research findings should be accompanied by guidance documents [34] and targeted technical support so stakeholders are able to enact evidence-based policy and programmatic changes. Dissemination of such evidence and guidance is a key component of targeted advocacy for decision-makers (a need cited by key informants), and such an effort should ensure a presence in key print publications, as well as online resources, as most of the interviewees noted that these are the resources that most influence their decision-making.

Interestingly, both global-level and country-level participants tended to defer accountability to others, electing not to assume responsibility themselves for promoting access to contraception as a core element of PMTCT. All of the participants acknowledged the role of family planning in HIV prevention, yet most simultaneously asserted that their professional focus lay elsewhere and that taking action to increase access to contraception for PMTCT was not within their current purview. At the same time, many interviewees mentioned the potential impact of having notable individuals–presumably people like themselves–champion the issue, serving as catalysts for an organized and multi-sector response.

It is also worth mentioning that although, perhaps surprisingly, none of the key informants highlighted this issue as a barrier, there is a critical need to improve the access to and quality of current national family planning programs (integrated or otherwise) in low- and middle-income countries. Particularly for low HIV prevalence areas, and in light of the fact that many women remain unaware of their HIV status in both low and high prevalence areas, we would argue that making improvements in family planning services for the *general population* may better contribute to meeting the contraceptive needs of HIV-positive women (and by extension reducing MTCT) than will integrating a poor quality family planning program with HIV services.

On a final note, the systematic stakeholder mapping approach we used filtered substantial amounts of information to arrive at a relatively small but comprehensive list (considering the volume of individuals working in HIV/AIDS) of professionally and geographically diverse individuals who play key roles in HIV/AIDS research, funding, policy and programming. Even the rigorous, systematic approach we used to identify individual stakeholders, however, has limitations and likely still missed “key players.” Others using this approach might consider supplementing their searches opportunistically–for example, by incorporating the tacit knowledge available through staff or from key informants, or by identifying a cohort via existing pre-vetted coalitions or working groups.

### Conclusion

Preventing unintended pregnancies in HIV-infected women is a proven PMTCT approach and therefore an essential component of a comprehensive PMTCT program. A diverse set of individuals have regional or international influence in the field of HIV, and the challenge of strengthening the implementation of contraception for HIV prevention is complex. Our findings affirm the existence of a multi-layered set of barriers and offer recommendations for addressing several of the primary challenges. Harnessing the influence of key individuals–to launch strengthened education, advocacy, and research efforts, as well as to support evolving funding mechanisms and policy structures–will be a necessary first step toward affecting measurable change.
